# Prediction of specific structural damage to the knee joint using qualitative isokinetic analysis

**DOI:** 10.1186/s12891-024-07434-w

**Published:** 2024-05-14

**Authors:** Feisheng Zheng, Rui Jia, Jinqun Ye, Mengyuan Li, Yunping Zhang, Guangqing Xu, Lei Zhang

**Affiliations:** 1 Department of Rehabilitation Medicine, Guangdong Provincial People’s Hospital (Guangdong Academy of Medical Sciences), Southern Medical University, Guangzhou, China; 2 Division of Joint Osteopathy and Traumatology, Center of Orthopedics Surgery, Guangdong Provincial People’s Hospital (Guangdong Academy of Medical Sciences), Southern Medical University, Guangzhou, China; 3 Clinical Medical College of Acupuncture moxibustion and Rehabilitation, Guangzhou University of Chinese Medicine, Guangzhou, China

**Keywords:** Isokinetic testing, Isokinetic moment curve, Anterior cruciate ligament, Meniscus, Patellofemoral joint

## Abstract

**Background:**

An isokinetic moment curve (IMC) pattern-damaged structure prediction model may be of considerable value in assisting the diagnosis of knee injuries in clinical scenarios. This study aimed to explore the association between irregular IMC patterns and specific structural damages in the knee, including anterior cruciate ligament (ACL) rupture, meniscus (MS) injury, and patellofemoral joint (PFJ) lesions, and to develop an IMC pattern-damaged structure prediction model.

**Methods:**

A total of 94 subjects were enrolled in this study and underwent isokinetic testing of the knee joint (5 consecutive flexion-extension movements within the range of motion of 90°-10°, 60°/s). Qualitative analysis of the IMCs for all subjects was completed by two blinded examiners. A multinomial logistic regression analysis was used to investigate whether a specific abnormal curve pattern was associated with specific knee structural injuries and to test the predictive effectiveness of IMC patterns for specific structural damage in the knee.

**Results:**

The results of the multinomial logistic regression revealed a significant association between the irregular IMC patterns of the knee extensors and specific structural damages (“Valley” - ACL, PFJ, and ACL + MS, “Drop” - ACL, and ACL + MS, “Shaking” - ACL, MS, PFJ, and ACL + MS). The accuracy and Macro-averaged F1 score of the predicting model were 56.1% and 0.426, respectively.

**Conclusion:**

The associations between irregular IMC patterns and specific knee structural injuries were identified. However, the accuracy and Macro-averaged F_1_ score of the established predictive model indicated its relatively low predictive efficacy. For the development of a more accurate predictive model, it may be essential to incorporate angle-specific and/or speed-specific analyses of qualitative and quantitative data in isokinetic testing. Furthermore, the utilization of artificial intelligence image recognition technology may prove beneficial for analyzing large datasets in the future.

## Introduction

As a valid and reliable test, isokinetic testing has become one of the gold standards for evaluating knee joint function in clinical practice and scientific research [1]. Quantitative data in isokinetic testing, such as peak torques, total work, and hamstring to quadriceps ratio (H: Q ratio, reflecting the ratio of peak flexion torque to peak extension torque), have been proven to be reliable in assessing the maximum strength, muscle endurance, and muscle imbalances of the knee [2]. However, these commonly used quantitative analyses may sometimes fail to characterize the dysfunction of different structural injuries. For example, in studies exploring the isokinetic characteristics of anterior cruciate ligament (ACL) rupture, meniscus (MS) injuries, and patellofemoral joint (PFJ) lesions, it is common to see similar results such as decreased peak torques, which do not distinguish the differences in dysfunction between the different injuries [2, 3]. In addition, these quantitative data from the isokinetic testing have also been used as predictors of future knee injuries while the conclusion remains inconsistent between studies [4, 5].

In recent years, an increasing number of researchers have become interested in the in-depth analysis of the isokinetic moment curve (IMC) as it provides a more precise assessment of joint function throughout the full range of motion [6, 7]. Different quantitative analysis techniques (such as statistical parametric mapping [8], discrete wavelet transform [9], principal component modeling [10], and arithmetic average [11]) have been applied in recent studies for in-depth analysis of IMC. These methods allow more detailed comparisons of torque at different angles as well as detailed variations in flexion-extension ratios, reducing the loss of information [12, 13]. Despite the robust utilization of quantitative data for evaluating knee joint strength, the qualitative characteristics of the curve representation, particularly the graphical representation of the IMC reflecting neuromuscular control during movement [14], are often overlooked [8, 15]. Neuromuscular adaptations and biomechanical alterations following knee injury may be important reasons for the irregular curve patterns in isokinetic testing, as presented in ACL rupture [16, 17], patellofemoral pain syndrome (PFPS) [18, 19], medial meniscus injury [14], and knee osteoarthritis [20].

As abnormal IMC patterns were found to be associated with specific knee injuries, several studies have attempted to use it as a predictor of knee-specific structural injuries and have shown a certain degree of reliability. Anderson et al. [21] qualitatively analyzed IMC in patients with PFPS using visual analysis and found that the positive and negative predictive values of abnormal patterns for the prediction of PFPS were 70% and 15%, respectively. Dauty et al. [22] performed isokinetic testing of knee extensors in 43 basketball players with a history of “jumper’s knee”. And an abnormal “Camel’s Back curve”, which may be secondary to a protective inhibitory mechanism, was demonstrated in the IMC of 35 players (81%) with a sensitivity and specificity of 81.3% and 100%, respectively. The possibility of using IMC patterns as auxiliary tools for making a differential diagnosis of knee injuries was also implied in the study by Iacono et al. [14]. Specifically, they found that knees with an ACL rupture exhibited a distinct “Shaking” pattern, which was absent in cases of isolated meniscus injuries or in healthy knees.

The potential use of IMC qualitative analysis (irregular IMC pattern) as a predictive tool for specific structural damage to the knee joint has been suggested in many studies [14, 21–23]. Physical examination is commonly used as a criterion for the initial diagnosis of knee injuries, however, its accuracy is highly dependent on the professionalism of the healthcare provider [24]. Moreover, isokinetic testing is more accessible than magnetic resonance imaging (MRI) or arthroscopy, and qualitative classification of IMC is relatively easier to perform and has proven to be reliable [14]. Thus, the development of an IMC pattern-damaged structure prediction model may be of considerable value in assisting the diagnosis of knee injuries in clinical scenarios.

However, only few studies have been conducted to develop relevant predictive models [21, 22]. Therefore, the aim of this study was, to explore the association between IMC pattern and specific structural damage in the knee (ACL rupture, MS injury, PFJ lesions including patellofemoral cartilage injury, and PFPS), and to test the accuracy of using IMC patterns as predictors of specific structural damage to the knee.

## Methods

### Subject enrollment

From September 2021 to September 2023, patients who complied with the inclusion criteria of our study were recruited into this study, which was as follows: (1) age between 18 and 55 years old. (2) 18< BMI<30. (3) MRI and physical examination suggested one of the following knee injuries: complete ACL rupture, MS tear (Grade III – abnormal high signal intensity in the central portion of the MS, extending to at least one articular surface) [25], patellofemoral joint (PFJ) lesion (≥ International Cartilage Repair society Grade III - cartilage lesions exceeding 50% of the depth) [26], or complete ACL rupture combined with MS tear (Grade III), and (4) no previous history of other knee injuries or surgeries. MRI interpretation and physical examination of all patients were performed by the same orthopedic surgeon (Mengyuan Li, co-author of this study). The exclusion criteria were as follows: (1) acute injury with significant swelling, (2) joint adhesions or significantly limited active range of motion and, (3) unable to complete isokinetic testing due to pain or other reasons. If necessary, patients underwent subsequent knee arthroscopy by the same specialized orthopedic surgeon to confirm the diagnosis of the damaged structures. In addition, for comparison with healthy knees, subjects with no history of knee injuries were recruited as the control group. This study was approved by the Ethics Committee of Guangdong Provincial People’s Hospital (KY-Z-2021-679-01) and complied with the Declaration of Helsinki. Written informed consent was obtained from all patients prior to enrollment.

### Isokinetic testing

To determine the association between IMC and specific injury structures and to establish an IMC pattern-damaged structure prediction model, isokinetic testing of the knee joint was performed in all included subjects using the ISOMED2000 isokinetic system (Basic System and Back System; D&R Ferstl GmbH, Hanau, Germany). To minimize further damage and the impact of acute pain on testing results, all subjects with knee injuries were ensured that their isokinetic testing was given at least three months after the occurrence of the injury. The test followed standardized procedures, and a strap was used to secure the participant’s trunk to reduce overcompensation. Before the test, patients were asked to perform five minutes of moderate-intensity cycling as a warm-up and to perform five familiarization tests before the official test. During the formal test, patients were asked to perform knee flexion and extension at an angular velocity of 60°/s with gravity correction applied and to repeat the maximal contraction five times in consecutive sessions within the range of motion (ROM) of 90°-10°. The angular velocities chosen in this study were aiming to obtain an IMC resulting from the maximum knee strength [27], and it was based on the settings of previous IMC studies [14, 28, 29]. Verbal encouragement was provided during the test, and peak torques and IMCs of the extensor muscle groups of the knee were recorded at the end of the test.

### Classification of IMC characteristics

**Fig. 1:**
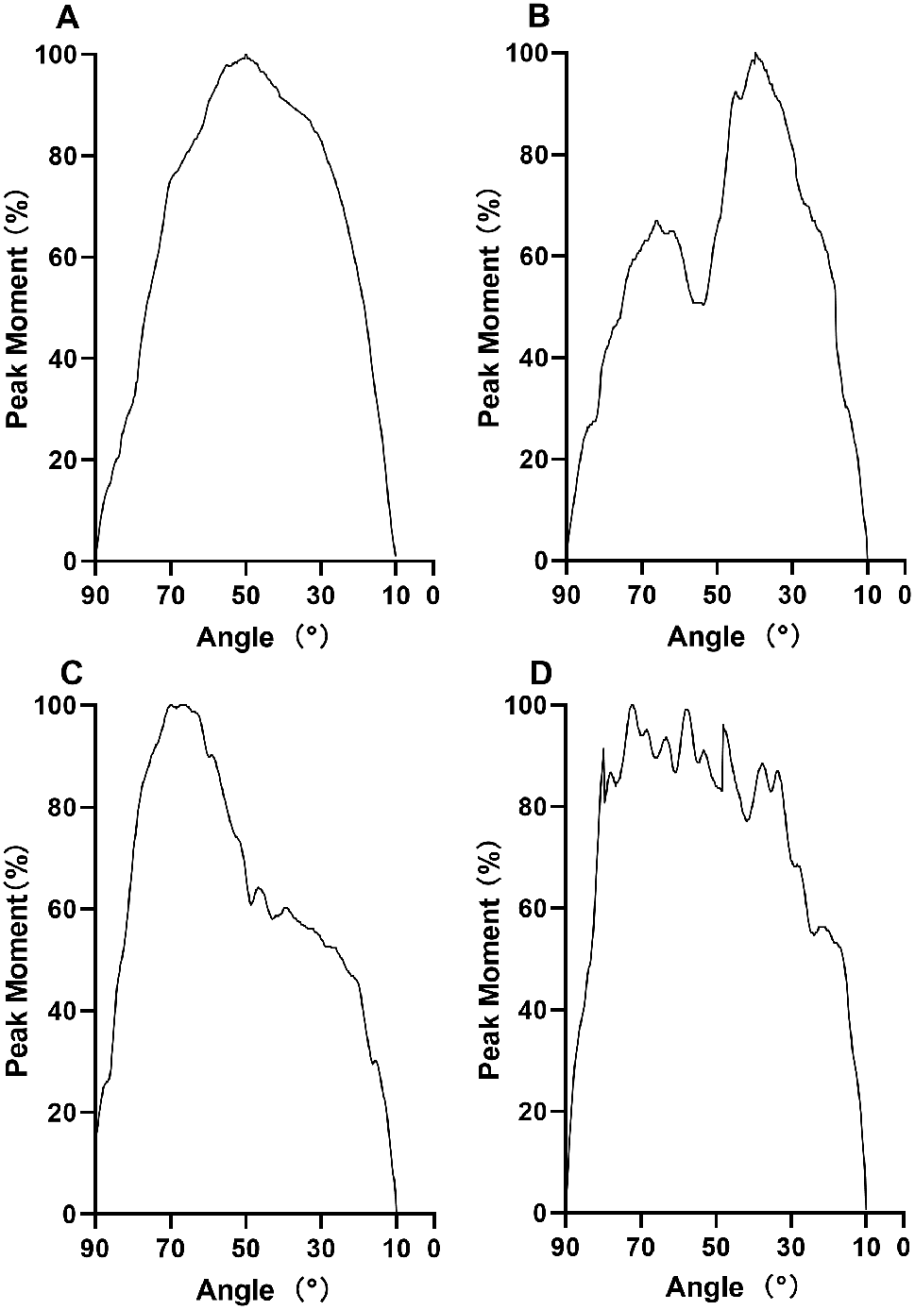
(**A**) “Normal” pattern of isokinetic moment curve for knee extensors. (**B**) “Valley” pattern of isokinetic moment curve for knee extensors. (**C**) “Drop” pattern of isokinetic moment curve for knee extensors. (**D**) “Shaking” pattern of isokinetic moment curve for knee extensors

The IMCs of the knee extensors were qualitatively analyzed by visual inspection based on previous studies. Using a linear interpolation technique, the IMCs were generated in steps of 1° [10]. Missing data (velocities less than 50°/s or missing angular data) were calculated by interpolation averaging [8]. The curves were normalized to peak moments before being presented to two blinded examiners, and to minimize the effects due to the change in movement strategy, only the 2nd, 3rd, and 4th repetitions were analyzed as described by Ayalon et al. [29]. The analysis of IMCs was based on the criteria of irregularity and consistency. Irregularity refers to the possible presence of breakpoints, which are defined as deviations of the IMCs from the prevailing pattern usually observed in the shape of the extension moment curve. Consistency refers to the number of repetitions in which the same irregularity occurred. Therefore, only irregular patterns shown in all three repetitions were considered consistent and representative of an abnormal IMC. Following these criteria, two blinded examiners qualitatively classified the specific patterns of IMCs based on the specific angles, shapes, amplitudes, and frequencies of the irregularities. According to the study by Iacono et al. [14], the breakpoint of IMCs analyzed by visual inspection led to the following classification of the curves:

1. Normal pattern: A continuous, smooth, interference-free curve, parabolic in shape, with peak value presenting near the midpoint of the curve. (Fig. 1A);
2. “Valley” pattern: A continuous and smooth curve with a major disturbance characterized by a break in the moment curve with a slight and sudden decrease in the torque output, occurring before or near the peak point. (Fig. 1B);
3. “Drop” pattern: A continuous and smooth curve with a major disturbance characterized by a break in the moment curve with a sharp and sudden decrease in the torque output, occurring right after the peak point. (Fig. 1C);
4. “Shaking” pattern: A curve with an irregular sequence, in the shape of an irregular flutter, appears along the middle of the moment curve including the peak point. (Fig. 1D).

Two examiners independently completed the first round of curve classification, followed by a second round of evaluation a week later. After a discussion of discrepancies, the third blinded examiner was invited to make a final decision for curves that could not be classified in agreement.

### Reliability analysis

Cohen’ s kappa coefficient (κ) was calculated to detect the reliability of the two examiners in classifying the 114 IMCs in the present study [30]. The agreement of the final IMC classification completed independently by each examiner and consistency of the same examiner’s IMC classification at one-week interval were used for inter-rater and intra-rater reliability calculations, respectively. According to Cohen et al. [31], κ ≤ 0 was defined as no agreement, 0.01–0.20 as none to slight, 0.21–0.40 as fair, 0.41– 0.60 as moderate, 0.61–0.80 as substantial, and 0.81–1.00 as almost perfect agreement.

### Statistical analysis

Demographic information of patients and healthy controls and the number of specific damaged structures are presented as mean ± standard deviation or n (%). Demographic differences were analyzed using independent t-test and chi-square test. A 5 × 4 contingency table was used to present the distribution of the four IMC patterns between different knee structural injuries and healthy controls. To investigate whether a specific abnormal curve pattern was associated with specific knee structural injuries and to test the predictive effectiveness of IMC patterns for specific structural damage in the knee, a multinomial logistic regression analysis was performed. The validity of the prediction model was evaluated based on its recall (equals to sensitivity), precision (positive predictive value), accuracy, F_1_ scores (the harmonic mean of precision and recall), and Macro-averaged F_1_ score (a simple average of the F_1_ scores over classes) [32]. All statistical analyses were conducted using SPSS 26 (SPSS Inc., Chicago, IL, USA) with a significance level of α < 0.05.

## Result

Five subjects were excluded from this study for the following reasons: limited ROM (*n* = 2), and unbearable pain during isokinetic testing (*n* = 3). In total, 94 subjects were enrolled in this study and divided into two groups, including the knee-injured group (*n* = 74; male: female = 44: 30; age: 39.89 ± 12.85 years; height: 167.27 ± 8.07 cm; body weight: 64.81 ± 12.54 kg), and the healthy control group (*n* = 20; male: female = 7: 13; age: 21.85 ± 1.60 years; height: 165.75 ± 7.77 cm; body weight: 58.95 ± 11.99 kg). There were no significant differences between groups in the demographic information except for age (*p*>0.05). All patients in the knee-injured group underwent MRI before isokinetic testing, and 25 patients underwent subsequent arthroscopic surgery. Diagnosed by the same, specialized orthopedic surgeon, the distribution of damaged knee structures in the patients was as follows: ACL (*n* = 10), MS (*n* = 17), PFJ (*n* = 24), and ACL + MS (*n* = 23). (Table 1).

**Table 1:** Demographic information and diagnosis of damaged structures

	Injured subjects (*n* = 74)	Control subjects (*n* = 20)
Sex (male: female)	44:30	7:13
Age (years)	39.89 ± 12.85	21.85 ± 1.60
Height (cm)	167.27 ± 8.07	165.75 ± 7.77
Weight (kg)	64.81 ± 12.54	58.95 ± 11.99
MRI taken	74 (100%)	
Underwent arthroscopy	25 (33.78%)	/
**Damaged structure**		
ACL	10 (13.51%)	/
MS	17 (22.97%)	/
PFJ	24 (32.43%)	/
ACL + MS	23 (31.08%)	/

Note: Data are reported as mean ± standard deviation or n (%). MRI: Magnetic resonance imaging; ACL: Anterior cruciate ligament; MS: Meniscus; PFJ: Patellofemoral joint; ACL + MS: Combined anterior cruciate ligament and meniscus injury

The distribution of IMC patterns for the involved leg of the injured subjects and bilateral legs of the control subjects is shown in Table 2. Classification of IMC characteristics reveals almost perfect inter-rater reliability (κ = 0.82) and intra-rater reliability (κ = 0.94) with 15 curves (13%) requiring the assistance of a third rater for definition.

**Table 2:** Distribution of IMC patterns for the involved leg of injured subjects and both legs of control subjects

Damaged structure		Injured subjects Control subjects *n* = 74				Control subjects *n* = 20
		ACL *n* = 10	MS *n* = 17	PFJ *n* = 24	ACL+MS *n* = 23	Healthy legs *n* = 40
IMC pattern	Normal pattern	2	4	4	3	35
	Valley pattern	2	1	10	6	2
	Drop pattern	3	1	1	8	1
	Shaking pattern	3	11	9	6	2

*Note*: The values represent the frequencies of four different irregular patterns that occurred in specific damaged structures. IMC: Isokinetic angle-specific moment curve; ACL: Anterior cruciate ligament; MS: meniscus; PFJ: Patellofemoral joint; ACL + MS: Combined anterior cruciate ligament and meniscus injury

The results of the multinomial logistic regression analysis (Table 3) show that abnormal IMC patterns of the knee were associated with specific structural injuries (*p* < 0.05). Compared to the normal IMC pattern, subjects exhibiting the “Valley” pattern had higher odds of having the following knee injuries: ACL, PFJ, and ACL + MS (*p* < 0.05). Subjects demonstrating a “Drop” pattern in IMC had higher odds of experiencing an ACL and ACL + MS injury compared to those who presented the normal pattern (*p* < 0.05). Compared to normal pattern, subjects with a “Shaking” IMC had increased odds of having any of the four injured knee structures included in our study (*p* < 0.05).

**Table 3:** Results of the multinomial logistic regression: odds ratio estimates for IMC patterns for diagnosed ACL, MS, PFJ, or ACL + MS injuries

Damaged structure	IMC pattern	Estimate	SE	OR	95%CI lower	95%CI Upper	p value
ACL							
	Valley	2.862	1.236	**17.500**	1.551	197.435	**0.021** ^*^
	Drop	3.961	1.365	**52.500**	3.620	761.444	**0.004** ^*^
	Shaking	3.268	1.167	**26.250**	2.665	258.516	**0.005** ^*^
Intercept		-2.862	0.727	NA	NA	NA	0.000
MS							
	Valley	1.476	1.334	4.375	0.320	59.726	0.268
	Drop	2.169	1.509	8.750	0.454	168.613	0.151
	Shaking	3.874	0.932	**48.125**	7.739	299.283	**0.000** ^*^
Intercept		-2.169	0.528	NA	NA	NA	0.000
PFJ							
	Valley	3.778	0.937	**43.750**	6.98	274.680	**0.000** ^*^
	Drop	2.169	1.509	8.750	0.454	168.613	0.151
	Shaking	3.673	0.943	**39.375**	6.199	250.092	**0.000** ^*^
Intercept		-2.169	0.528	NA	NA	NA	0.000
ACL + MS							
	Valley	3.555	1.014	**35.000**	4.795	255.472	**0.000** ^*^
	Drop	4.536	1.219	**93.333**	8.552	1018.547	**0.000** ^*^
	Shaking	3.555	1.014	**35.000**	4.795	255.472	**0.000** ^*^
Intercept		-2.457	0.602	NA	NA	NA	0.000

IMC: Isokinetic moment curve; SE: Standard error; OR: Odds ratio; CI: Confidence interval; ACL: Anterior cruciate ligament; MS: Meniscus; PFJ: Patellofemoral joint; ACL + MS: Combined anterior cruciate ligament and meniscus injury; NA: not applicable; * Indicates p value from a Wald test statistic < 0.05

Table 4 shows the recall, precision, and F_1_ score of the regression model for predicting each of the following four knee structural injuries and healthy knees. The Macro-averaged F_1_ score, and the overall accuracy of this prediction model were 0.426 and 56.1%, respectively. More specifically, the predictive efficacy of this model for the four specific knee structural injuries was as follows: 0-64.7% for recall, 0-57.1% for precision, and 0-0.458 for F_1_ score.

**Table 4:** Recall, Precision, and F_1_ score for five different dependent variables and Macro-averaged F_1_ score and accuracy of the multinomial logistic regression: IMC pattern as a predictor for specific damaged structures in the knee

Actual damaged structure	Predicted damaged structure										
		ACL	MS	PFJ	ACL+ MS	Healthy	Recall		Precision		F_1_ score
ACL		0	3	2	3	2	0		0		0
MS		0	11	1	1	4	64.7%		35.5%		0.458
PFJ		0	9	10	1	4	41.7%		47.6%		0.444
ACL + MS		0	6	6	8	3	34.8%		57.1%		0.432
Healthy		0	2	2	1	35	87.5%		72.9%		0.795
Macro-averaged F_1_ score											0.426
Accuracy											56.1%

ACL: Anterior cruciate ligament; MS: Meniscus; PFJ: Patellofemoral joint; ACL + MS: Combined anterior cruciate ligament and meniscus injury

## Discussion

Through qualitative visual analysis of knee IMC in four types of knee structural injuries and healthy controls, the purpose of this study was to investigate the association between abnormal IMC patterns and specific knee structural injuries and validate its accuracy as a tool for predicting specific knee structural injuries. Despite the predictive efficacy and reliability of the developed prediction model was relatively low. The results of our study revealed a significant association between the specific structural damage and its irregular IMC patterns of the knee extensors (“Valley” - ACL, PFJ, and ACL + MS, “Drop” - ACL, and ACL + MS, “Shaking” - ACL, MS, PFJ, and ACL + MS), which provides a basis for further research on the biomechanical mechanisms behind the specific dysfunctional features of different knee injuries in the future.

Specific biomechanical and neuromuscular control alteration accompanied by different structural injuries of the knee play an essential role in the aberrant changes in its IMC characteristics [33], and thus give the qualitative analysis of IMC the potential to reflect specific dysfunctional profiles and to predict knee injuries. In the present study, patients with ACL, PFJ, and ACL + MS injuries presented an abnormal IMC pattern (“Valley”) characterized by sudden interruptions in smoothness. Similar results have been reported in other studies that target in isokinetic testing of PFJ-related lesions [21, 34, 35]. In addition, the importance of eccentric IMC of the quadriceps muscle with a break point has also been reported. When considered as a predictor of PFPS, it has a positive predictive value of up to 70% [21]. Although a different isokinetic test (eccentric strength) was used in the aforementioned studies, its hypothesis regarding the reason for the occurrence of breaks in the IMC may also be applicable to the present study: The presence of breakpoints may be a neuromuscular adaptive strategy used by PFPS patients to reduce pain in a particular range of motion. This is achieved by decreasing the degree of quadriceps contraction to reduce the level of stress on the peripatellar retinacular supports, joint capsule, synovium, and PFJ cartilage [36]. In the current study, ACL deficit was also demonstrated to be significantly associated with the presence of a “Valley” pattern of IMC (*p* > 0.05). And it is likely to be associated with the underlying PFJ impairment in some of the patients, as ACL deficiency was proven to be a risk factor for PFJ osteoarthritis [37–39]. Despite being speculative, this result suggests that quantitative IMC analysis may be useful in identifying specific injury structures that have affected the biomechanical function of the knee, although not yet detected in imaging.

Analysis of the IMC characteristics of patients with ACL related injuries revealed its unique association with the “Drop” pattern compared to other types of injuries, which is consistent with the previous studies [40, 41]. Several studies on ACL injuries have consistently concluded that significant quadriceps peak torque deficits were only found when the knee was at a flexion angle of less than 40°-45° during concentric isokinetic movements at 60°/s [11, 42, 43], which is highly similar to the manifestation of the “Drop” pattern. The occurrence of the “Drop” pattern (manifested by a sudden and rapid drop in the moment curve near the peak value) is likely to be associated with specific biomechanical changes following ACL injury. It was reported that compared to healthy knees, significantly greater anterior tibial translation was presented in ACL-deficient knees when performing loaded open-chain extension in the range of 64° to 10° [44, 45]. Thus, motion in this range may have overstretched the secondary restraints of the knee due to the lack of anterior stability, which in turn leads to dysfunction of the knee extensors shown in the IMC [14]. The results of previous studies suggest that the interpretation of IMC graphic features may be of great importance in the prediction of ACL injuries.

Our findings suggest that a “Shaking” IMC may be a common dysfunctional feature of the four structural knee injuries included in this study. During the curve classification process, it was found that IMCs with “Shaking” characteristics not only exhibit reduced smoothness in the mid-section of the curve image but also demonstrated abnormalities in the absolute value of the peak extension torques and the H: Q ratio. Patients with “Shaking” IMCs generally had significantly lower peak extension torques than other subjects and their H: Q ratios were close to or greater than 1 (the average H: Q ratio in 60°/s was 0.65 ± 0.12 in healthy populations) [46]. The anomalous features of these quantitative data also played a role in the classification of the curves, as 10 curves were difficult to distinguish between “Valley” and “Shaking” by the IMC features alone. Such qualitative and quantitative characterization of the “Shaking” pattern suggests the presence of a wide range of strength reduction during movement as well as abnormal muscle coordination, which might result from neuroadaptive mechanisms such as quadriceps dyskinesia and extra hamstring co-contractions following knee injury following knee injuries [47, 48]. However, our findings were somewhat different from those of a previous study, as an absence of “Shaking” patterns in subjects with MS injuries was reported. In this study, we included subjects with grade III meniscal tears (both medial and lateral), whereas the study by Iacono et al. [14] only included patients with medial meniscal injuries and did not specify the severity of the injuries. We therefore consider that the difference in the severity and region of injury may be one of the potential reasons for the discrepancy between the results. Incorporating the surface electromyography technique in future isokinetic test studies will help to better explain the biomechanical mechanisms behind the different abnormal IMCs.

Furthermore, our study developed a predictive model for knee injuries using the qualitative characteristics of IMC as a predictor. Multinomial logistic regression analysis showed that irregular IMC patterns were statistically significant (*p* < 0.05) in predicting specific damaged structures in the knee. However, the accuracy and Macro-averaged F_1_ score of the model were 56.1% and 0.426, respectively, indicating relatively low predictive efficacy and reliability. Besides, the 0 score for recall, precision, and F1 score when predicting ACL rupture, suggesting the potential challenge of predicting ACL injury alone using the method utilized in the present study, despite the observations of various IMC patterns in individuals with ACL injuries.

This study, however, is subject to several limitations. First, there was a statistical difference in age between the two groups, though this may have had little effect on the conclusions of this study. As the main components analyzed in this study were the characteristics of the IMC, while not the absolute value of the peak torque associated with age. Second, absence of imaging of the control knee may have resulted in incorrect grouping, despite most of the control subjects in this study demonstrating a normal IMC pattern. However, the impact of such potential grouping errors may be magnified in future large-sample studies, and therefore this is a point of great need for improvement. Besides, the imbalance of samples among the groups due to the insufficient overall sample size, especially the excessively small sample size in the isolated ACL injury group, may be an essential factor that responsible for the low predictive efficacy of the model. The pain intensity experienced by the subjects and the specific angle at which it appeared during the isokinetic testing are also important factors to be considered in further studies. As the pain level has been proven to be associated with peak torque reduction especially during extension movements [49], and the specific angle of pain occurrence may help to further analyze the biomechanical mechanisms behind the abnormal IMC. For future research, extracting IMC features of specific knee injuries from a large number of IMC using deep learning related algorithms [50], while incorporating essential angle-specific and/or speed-specific quantitative data from isokinetic testing in the building of the predictive model might be a promising approach [51, 52].

## Conclusion

In conclusion, associations between irregular IMC patterns and specific knee structural injuries were identified (“Valley” - ACL, PFJ, and ACL + MS, “Drop” - ACL, and ACL + MS, “Shaking” - ACL, MS, PFJ, and ACL + MS). However, the accuracy and Macro-averaged F_1_ score of the established predictive model indicated its relatively low predictive efficacy. For the development of a more accurate predictive model, it may be essential to incorporate angle-specific and/or speed-specific analyses of qualitative and quantitative data in isokinetic testing. Furthermore, the utilization of artificial intelligence image recognition technology may prove beneficial for analyzing large datasets in the future.

## Data Availability

The datasets used in this study are available upon request from the first or corresponding author. Access to the data is subject to any applicable ethical and legal approvals.

## References

[1] Kambič T, Lainščak M, Hadžić V (2020). Reproducibility of isokinetic knee testing using the novel isokinetic SMM iMoment dynamometer. PLoS ONE.

[2] Urhausen AP (2022). Measurement properties for muscle strength tests following anterior cruciate ligament and/or meniscus injury: what tests to use and where do we need to go? A systematic review with meta-analyses for the OPTIKNEE consensus. Br J Sports Med.

[3] Stefanik JJ (2011). Quadriceps weakness, patella alta, and structural features of patellofemoral osteoarthritis. Arthritis Care Res (Hoboken).

[4] Green B, Bourne MN, Pizzari T (2018). Isokinetic strength assessment offers limited predictive validity for detecting risk of future hamstring strain in sport: a systematic review and meta-analysis. Br J Sports Med.

[5] Kellis E, Sahinis C, Baltzopoulos V (2023). Is hamstrings-to-quadriceps torque ratio useful for predicting anterior cruciate ligament and hamstring injuries? A systematic and critical review. J Sport Health Sci.

[6] Tracy BL, Enoka RM. *Older adults are less steady during submaximal isometric contractions with the knee extensor muscles* J Appl Physiol (1985), 2002 10.1152/japplphysiol.00954.2001. 10.1152/japplphysiol.00954.2001 11842033

[7] Solomonow M (1987). The synergistic action of the anterior cruciate ligament and thigh muscles in maintaining joint stability. Am J Sports Med.

[8] Baumgart C (2018). Angle-specific analysis of isokinetic quadriceps and hamstring torques and ratios in patients after ACL-reconstruction. BMC Sports Sci Med Rehabil.

[9] Czaplicki A et al. Using the discrete wavelet transform in assessing the effectiveness of rehabilitation in patients after ACL reconstruction. Acta Bioeng Biomech, 2017. 29205225

[10] Almosnino S (2014). Principal component modeling of isokinetic moment curves for discriminating between the injured and healthy knees of unilateral ACL deficient patients. J Electromyogr Kinesiol.

[11] Huang H (2017). Isokinetic angle-specific moments and ratios characterizing hamstring and quadriceps strength in anterior cruciate ligament deficient knees. Sci Rep.

[12] Warmenhoven J (2018). A force profile analysis comparison between functional data analysis, statistical parametric mapping and statistical non-parametric mapping in on-water single sculling. J Sci Med Sport.

[13] Pataky TC, Vanrenterghem J, Robinson MA (2015). Zero- vs. one-dimensional, Parametric vs. non-parametric, and confidence interval vs. hypothesis testing procedures in one-dimensional biomechanical trajectory analysis. J Biomech.

[14] Iacono AD (2018). Isokinetic moment curve abnormalities are associated with articular knee lesions. Biol Sport.

[15] Alhammoud M (2019). Discipline and Sex Differences in Angle-Specific Isokinetic Analysis in Elite skiers. Int J Sports Med.

[16] Herrington L, Turner M, Horsley I (2003). The relationship between ACL deficiency, functional performance and a break in the isokinetic moment curve of the knee flexors. Isokinet Exerc Sci.

[17] Ikeda H, Kurosawa H, Kim S-G. Quadriceps torque curve pattern in patients with anterior cruciate ligament injury. International orthopaedics; 2002. 10.1007/s00264-002-0402-0 PMC3620983 12466872

[18] Dvir Z, et al. Quadriceps function and patellofemoral pain syndrome. Part I: pain provocation during concentric and eccentric isokinetic activity. Isokinetics and Exercise Science; 1991.

[19] Herrington L, Williams S, George K. The relationship between arthroscopic findings and isokinetic quadriceps performance in patellofemoral pain syndrome patients: an initial investigation. Res Sports Medicine: Int J, 2003.

[20] Kuijt M-TK, et al. Knee and ankle osteoarthritis in former elite soccer players: a systematic review of the recent literature. Journal of science and medicine in sport; 2012. 10.1016/j.jsams.2012.02.008 22572082

[21] Anderson G, Herrington L (2003). A comparison of eccentric isokinetic torque production and velocity of knee flexion angle during step down in patellofemoral pain syndrome patients and unaffected subjects. Clin Biomech (Bristol Avon).

[22] Dauty M (2019). Jumper’s knee mechanical consequences in professional basketball players: the Camel’s back curve. Eur J Appl Physiol.

[23] Afzali L (1992). A new method for the determination of the characteristic shape of an isokinetic quadriceps femoris muscle torque curve. Phys Ther.

[24] Peeler J, Leiter J, MacDonald P (2010). Accuracy and reliability of anterior cruciate ligament clinical examination in a multidisciplinary sports medicine setting. Clin J Sport Med.

[25] Li CA (2013). Correlation of histological examination of meniscus with MR images: focused on high signal intensity of the meniscus not caused by definite meniscal tear and impact on mr diagnosis of tears. Korean J Radiol.

[26] Shah AJ, Patel D (2021). Imaging update on cartilage. J Clin Orthop Trauma.

[27] Janicijevic D (2020). Isokinetic testing: sensitivity of the force-velocity relationship assessed through the two-point method to discriminate between muscle groups and participants’ physical activity levels. Int J Environ Res Public Health.

[28] Ayalon, et al. Identification of feigned strength test of the knee extensors and flexors based on the shape of the isokinetic torque curve. Isokinetics & Exercise Science; 2001.

[29] Ayalon M, Barak Y, Rubinstein M. Qualitative analysis of the isokinetic moment curve of the knee extensors. Isokinetics and exercise science; 2002.

[30] McHugh ML. Interrater reliability: the kappa statistic. Biochem Med (Zagreb); 2012. PMC3900052 23092060

[31] Cohen J. A coefficient of agreement for nominal scales. Educational and psychological measurement; 1960.

[32] Takahashi K (2022). Confidence interval for micro-averaged F (1) and macro-averaged F (1) scores. Appl Intell (Dordr).

[33] Sjölander P, Johansson H, Djupsjöbacka M (2002). Spinal and supraspinal effects of activity in ligament afferents. J Electromyogr Kinesiol.

[34] Dvir Z, Halperin N (1992). Patellofemoral pain syndrome: a preliminary model for analysis and interpretation of isokinetic and pain parameters. Clin Biomech (Bristol Avon).

[35] Herrington L, Williams S, George K (2003). The relationship between arthroscopic findings and isokinetic quadriceps performance in Patellofemoral Pain Syndrome patients: an initial investigation. Res Sports Med.

[36] Goodfellow J, Hungerford DS, Zindel M (1976). Patello-femoral joint mechanics and pathology. 1. Functional anatomy of the patello-femoral joint. J Bone Joint Surg Br.

[37] Culvenor AG (2013). Is patellofemoral joint osteoarthritis an under-recognised outcome of anterior cruciate ligament reconstruction? A narrative literature review. Br J Sports Med.

[38] Culvenor AG, Crossley KM (2016). Patellofemoral Osteoarthritis: are we missing an important source of symptoms after Anterior Cruciate Ligament Reconstruction?. J Orthop Sports Phys Ther.

[39] Huang W (2020). Prevalence of patellofemoral joint osteoarthritis after anterior cruciate ligament injury and associated risk factors: a systematic review. J Orthop Translat.

[40] Ikeda H, Kurosawa H, Kim SG (2002). Quadriceps torque curve pattern in patients with anterior cruciate ligament injury. Int Orthop.

[41] Stratford P (1987). Diagnostic value of knee extension torque tracings in suspected anterior cruciate ligament tears. Phys Ther.

[42] Shirakura K, Kato K, Udagawa E (1992). Characteristics of the isokinetic performance of patients with injured cruciate ligaments. Am J Sports Med.

[43] Eitzen I (2010). Anterior cruciate ligament-deficient potential copers and noncopers reveal different isokinetic quadriceps strength profiles in the early stage after injury. Am J Sports Med.

[44] Yack HJ, Collins CE, Whieldon TJ (1993). Comparison of closed and open kinetic chain exercise in the anterior cruciate ligament-deficient knee. Am J Sports Med.

[45] Norouzi S (2013). Rehabilitation after ACL injury: a fluoroscopic study on the effects of type of exercise on the knee sagittal plane arthrokinematics. Biomed Res Int.

[46] Baroni BM (2020). Hamstring-to-quadriceps Torque Ratios of Professional Male Soccer players: a systematic review. J Strength Cond Res.

[47] Solomonow M, Krogsgaard M (2001). Sensorimotor control of knee stability. A review. Scand J Med Sci Sports.

[48] Pamukoff DN (2017). Quadriceps function and Hamstrings Co-activation after Anterior Cruciate Ligament Reconstruction. J Athl Train.

[49] Solà R, et al. Isokinetic assessment of muscle strength in patients with knee pain. Osteoarthritis and Cartilage; 2012.

[50] Khurshid S (2022). ECG-Based deep learning and clinical risk factors to Predict Atrial Fibrillation. Circulation.

[51] Read PJ (2022). Angle specific analysis of hamstrings and quadriceps isokinetic torque identify residual deficits in soccer players following ACL reconstruction: a longitudinal investigation. J Sports Sci.

[52] Sousa LA (2019). Modeling the Angle-Specific Isokinetic hamstring to quadriceps ratio using Multilevel Generalized Additive models. Med (Kaunas).

